# Clinical and epidemiological profile of cleft lip and palate patients in Peru, 2006 – 2019

**DOI:** 10.4317/jced.58976

**Published:** 2021-11-01

**Authors:** Claudio Peña-Soto, Luis-Ernesto Arriola-Guillén, Antonio Díaz-Suyo, Javier Flores-Fraile

**Affiliations:** 1 PhD, Associate Professor of Faculty of Sciences of Life and Health, School of Dentistry, Universidad Científica del Sur, Lima, Peru; 2 PhD, Associate Professor Division of Orthodontics, School of Dentistry, Universidad Científica del Sur, Lima, Peru; 3 MsC, Professor of Faculty of Sciences of Life and Health, School of Dentistry, Universidad Científica del Sur, Lima, Peru; 4 PhD, Professor of Department of Surgery, University of Salamanca, Instituto de Investigación Biomédica de Salamanca (IBSAL), Salamanca, Spain

## Abstract

**Background:**

The purpose of this study was to determine the clinical-epidemiological profile of patients with cleft lip and / or palate in Peru from 2006 to 2019.

**Material and Methods:**

This retrospective and cross-sectional study analyzed 3,923 patients with cleft lip and palate attended by surgical missions of the Operación Sonrisa Perú from January 2006 to December 2019. The clinical profile of the patients treated included: type of cleft (cleft lip CL, cleft palate CP, cleft lip and palate CLP and submucosal SM), surgery performed (cheiloplasty, palatoplasty, cleft rhinoplasty, fistula repair, pharyngeal flap), surgical time according to number of interventions. Likewise, affiliation variables such as sex, age and birthplace were recorded. Descriptive analysis was performed. Associations were determined using the Pearson’s Chi-square test and Two-sample test of proportions were used for comparing the percentages during time. A *p* value <0.005 was considered significant.

**Results:**

The most frequent diagnosis was CP (n = 1411, 35.97%). We identified a statistically significant association between the diagnosis of CL, CP and gender (*p* = 0.045), being more prevalent in males. A higher prevalence of CL was also observed on the left side and in males (n = 183). Cheiloplasty was the most frequent first surgical intervention performed (n = 837, 47.42%) followed by fistula repair as the second intervention (n = 428, 42.29%).

**Conclusions:**

Cleft lip and palate are more frequent in males, with CP being the most frequent. CL is more frequent on the left side and the first surgical approach in these patients is lip closure.

** Key words:**Epidemiology, cleft lip and palate, surgical missions.

## Introduction

Cleft lip and palate are the most frequent congenital structural deficiencies in the maxillofacial region due to lack of union between some facial processes during embryonic development (1). There are many classifications for this clinical picture with one involving classification according to the exposed anatomy: involvement of the lip is considered as unilateral (right or left) or bilateral cleft lip and is classified as incomplete or complete if nasal tissues are involved. Complete or incomplete cleft palate (one third or two thirds) or cleft lip and palate is defined when the condition presents bilateral or unilateral involvement of the palate and the lip. Involvement of all three structures is defined as a naso-lip-palatal cleft (2).

Patients with cleft lip and palate (CLP) present significant morbidity along their lifetime. In addition to facial manifestations, these conditions cause disorders when speaking, hearing, chewing, swallowing and breathing (3). According to the data available, the prevalence of CLP is approximately 1 in 700 children born worldwide, with cleft lip (CL), cleft palate (CP), or both (1). There are considerable variations according to ethnicity and geographical location, with the highest prevalence being found among Asians and Native Americans (1/500) while Caucasian, Hispanic and Latino populations have a medium prevalence (1/1000) and the lowest is found in the African population (1/2500) (4,5).

The treatment of CLP reflects health inequality in the modern world. The evidence available suggests that in the absence of any intervention, neonatal mortality is very high, except in the case of slight defects (6). In Latin America, one in ten children with CLP dies within the first year, and thus, organizations such as Operation Smile have been working for the well-being of patients with CLP around the world (7). This organization has been operating in Peru for 20 years with approximately 6,000 patients having been treated in more than 100 missions in different areas of the country. Knowledge of the prevalence, sex, nutritional status and distribution according to geographic region will help to plan future actions to improve care (8). In this context, the purpose of this study was to determine the epidemiological and clinical profile of patients treated by Operación Sonrisa Perú from 2006 to 2019.

## Material and Methods

This was a cross-sectional, retrospective study based on medical records. The information was obtained by analyzing data from 78 missions of Operation Smile throughout Peru from January 2006 to December 2019. To obtain and analyze the information, authorization was obtained from the corresponding entities of Operación Sonrisa Perú and the Institutional Committee for Research Ethics of Científica del Sur University, Lima - Peru (001-2020-PRO99). The characteristics of the patients were captured from a common database from the medical records which recorded the patient’s basic data as age, sex, birthplace, surgery performed and time of the surgery; we used the information available from all the patients attended during the 2006-2019 period.

To obtain the epidemiological profile of the treated patients, we analyzed the variables of sex, age and birthplace. The clinical profile included the following diagnostic variables: cleft lip (CL), cleft palate (CP), cleft lip and palate (CLP) and submucosal (SM), surgery performed: cheiloplasty, palatoplasty, cleft rhinoplasty, fistula repair and pharyngeal flap, time of the surgery (first or second intervention).

-Statistical Analysis 

We used the statistical program STATA 16.0 for data analysis. The descriptive analysis of categorical variables was evaluated using frequencies and percentages. For descriptive analysis of numerical variables, mean, median, standard deviation, variance, minimum and maximum number were used. When analyzing the association between diagnostic and gender, the Pearson’s Chi square test was used. For comparing the percentages during time between the diagnosis and the part of the face affected in males and females Two-sample test of proportions were used. The level of significance was established at 5%.

## Results

A total of 3923 patients were included in the study. The largest number of patients was 0 to 5 years old (n = 2639, 67.27%). The patients ranged in age from 15 days to 69 years, with a mean of 5.85 ± 8.32 years and a median of 2 years. Most of the cases were males (n = 2341, 59.67%) compared to females (n = 1582, 40.33%). In relation to region in which the patients reside in Peru, most were from region 5 (Lima-Callao) (n = 1269, 32.35%), followed by region 1 (Tumbes, Piura and Lambayeque) (n = 618, 15.75%) and region 9 (Arequipa) (n = 587, 14.96%) (Table 1).

A statistically significant association was found between diagnosis and sex (*p* = 0.045). CL was present in 59.50% of males and 40.5% of females. CP and CLP were more frequent in males compared to females (57.62% vs. 42.38% and 61.12% vs. 38.58%, respectively. In general, CP was the most frequent diagnosis (n = 1411, 35.97%), followed by CLP (n = 1255, 31.99%) and CL (n = 1158, 29.52%), while submucosal fissure (SM) was the least frequent diagnosis (n = 99, 2.52%) (Table 2).

**Table 1:**
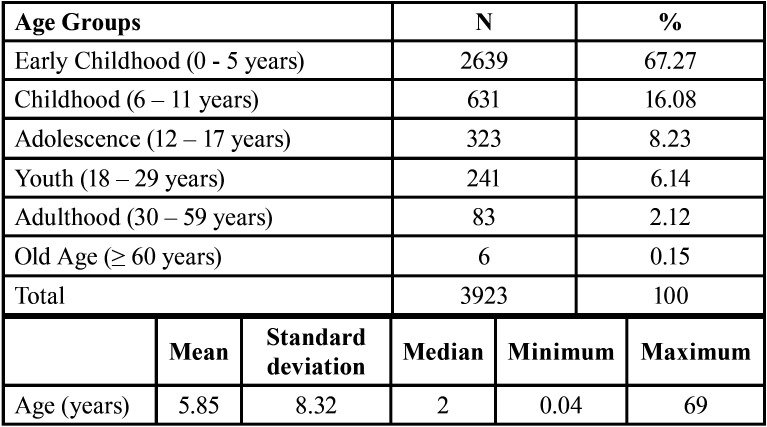
Distribution of age groups of the patients evaluated.

**Table 2:**
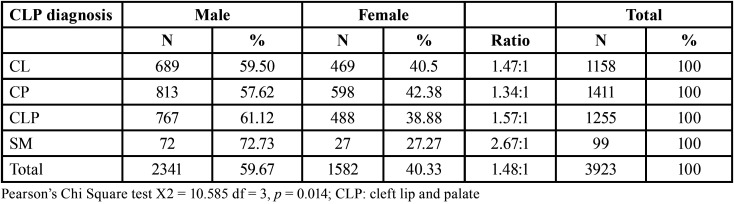
Association between the diagnosis of cleft lip and palate and sex.

In the analysis of the relationship between the diagnosis, the part of the face affected and sex, the prevalence of CL was higher on the left side and in males (n=183). Bilateral cleft and male sex were the most frequent in CP, CLP and SM. We compared the percentages in males and females and found a statistically significant differences in the CL of the left side and all the diagnoses of bilateral and right-sided affection (Table 3).

**Table 3:**
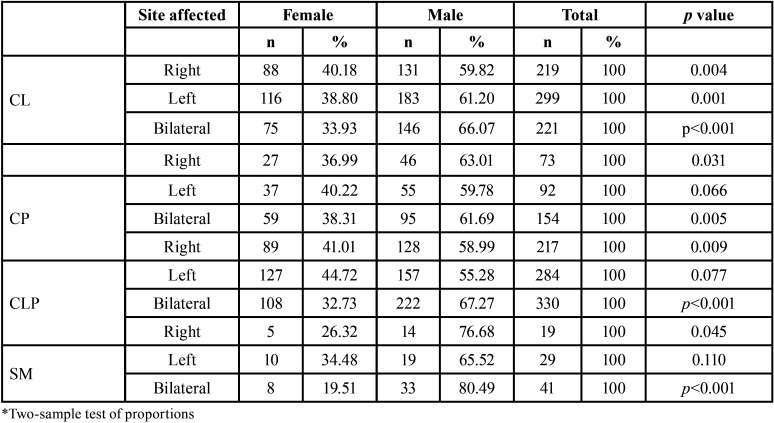
Evaluation of the relationship between diagnosis, side of face affected and gender of the patients.

Regarding the surgical procedure, the most frequent first interventions were cheiloplasty and palatoplasty. Cheiloplasty was performed in 837 patients (47.42%), 320 women and 517 men with a median age of 0.5 years. Palatoplasty was performed in 689 patients (39.04%), 277 females and 412 males with a median age of 2 years for females and 1 year for males. In patients who undergo a second intervention, the most interventions performed were fistula repair and cheiloplasty. Fistula repair was performed in 428 patients (42.29%), 174 females and 254 males with a median age of 6 years for both sexes. Cheiloplasty was performed in 273 patients (26.98%), 106 females and 167 males with a median age of 8 years for both females and males (Table 4).

**Table 4:**
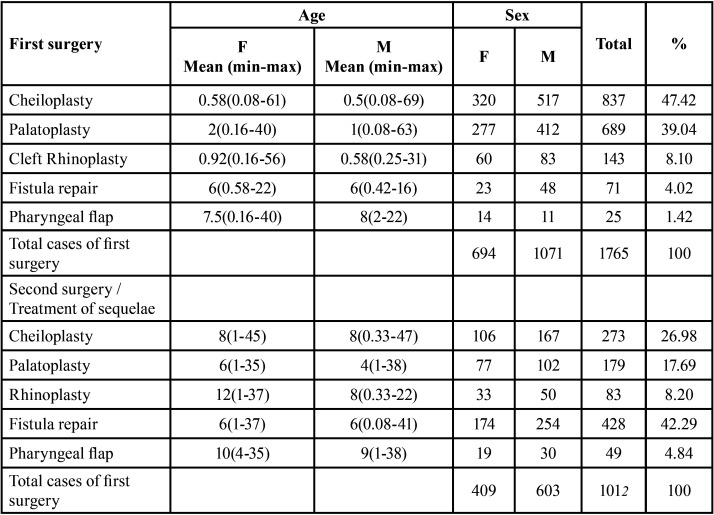
Interventions performed and surgical time according to age and gender.

## Discussion

The first step for planning oral health services is knowledge of the profile of the target population (9). Based on this knowledge, different preventive and curative strategies can be planned.

The surgical missions of Operación Sonrisa Perú in the period from 2006 to 2019 were developed in the Peruvian departments of Arequipa, Ayacucho, Cusco, Iquitos, Lima, Piura, Puno and Trujillo. However, due to the health service needs of people at a national level, there is a registry of patients of all the departments and regions of Peru (Table 1).

Our study revealed that cleft defects are more frequent in males, similar to other studies conducted in India (6,10), Brazil (11), Kenya (12), Iran (13,14), Saudi Arabia (15), Japan (16) and Peru (17,18).

In the study population, the most frequent diagnosis was CP (n = 1411, 35.97%), similar to what was reported by Blanco-Davila *et al*. (19) who evaluated the incidence of cleft lip and palate in a 10-year retrospective study in Mexico. The next most frequent diagnoses were CLP (n = 1255, 31.99%) and CL (n =1158, 29.52%) with the frequency of the 3 diagnoses being CP> CLP> CL, which is the same as that described by Belliss and Gemuth (20) in a 20-year retrospective study in Scotland. Sah and Powar (8) found the frequency to be CLP> CL> CP in a 5-year study in India while Nagase *et al*. (16) reported CLP> CP> CL in a 12-year study in Japan. The frequency of the 3 diagnoses was higher in males with a ratio of 1.3:1 for CP, 1.5:1 for CLP and 1.5:1 for CL. Sah and Powar (8) also reported a higher frequency in males with ratios of 1.1:1 for CP, 1.2:1 for CLP, and 1.3:1 for CL, and Kim *et al*. (21) found a ratio of 2.5:1 for CLP and 2.1:1 for CL.

In our study, CL was higher in males (60.3% lip - 61.4% lip and palate) compared to females (39.6% lip - 38.5% lip and palate), with a 1.5:1 ratio. These data coincide with those reported by Marazita (22), Dixon *et al*. (4), and Parada *et al*. (5) who described a higher incidence of lip involvement in males with a ratio of 2:1.

In relation to involvement of only the palate, this was more frequent in males (57.6%) compared to females (42.3%) with a 1.3:1 ratio and coincides with the results of Pons-Bonals *et al*. (2), who reported greater involvement of the palate in males (58%) compared to females (27%) with a ratio of 2.1:1. However, these results differ from those of Marazita (22), Dixon *et al*. (4), and Parada *et al*. (5) who reported a higher presentation of palate involvement in females with a ratio of 2:1.

Several studies have described that CL is more frequent on the left side and in males (2,19, Mahdi *et al*., 2013.) According to the results of our study, the percentage of left CL in males and females was 61.2% and 38.8% respectively, and are therefore, in agreement with previously published data. However, the mechanism of this higher frequency is unclear. Hirayama (23) studied human fetuses in late pregnancy and observed slower development of the facial artery at that site of the disease. However, Yorita (24) suggested an association between cleft laterality and handedness, although this has not been consistently demonstrated (25-27).

With respect to the surgical approach, regardless of the degree of tissue involvement, the surgical principle remains the same: attempt to normalize both anatomy and physiology, as well as improve the psychological effects of these conditions on the patient (28). The number of interventions needed, and when to perform the first surgery have been largely debated. Most cleft lip and palate care centers schedule surgery at between 10 to 12 weeks of age, following the rule of 10, which suggests that the infant must be at least 10 weeks old, weigh at least 10 pounds and have hemoglobin values of 10 mg/dl before lip repair (29). In the present study, a total of 1,765 patients underwent their first surgery at a median age of 0.5 years in both sexes and the most frequently surgery was cheiloplasty (n = 837, 47.4%). These results agree with the established by international organizations for the care of patients with cleft lip and palate, with the aim of the first intervention being closure of the lip (2,30,31).

The missions of Operation Smile Peru have always had the main objective of providing safe and quality surgery to children who need it, however throughout the years the patterns in health services have been changing, since 2013 the approach has been more interdisciplinary with the participation of orthodontists and speech therapists in decision-making for the treatments carried out.

There is general agreement that surgical treatment of these cases requires more than one intervention. The guidelines of the Mexican Ministry of Health for the prevention, treatment and rehabilitation of children with CL and / or CP propose the need for at least four interventions to achieve adequate repair (32) On the other hand, in a survey among the members of the Euro Cleft Project (30), the total number of surgeries deemed necessary to close the cleft varied from one: 10 (5%), two: 144 (71, 1%), three: 43 (21.9%) and four: 4 (2%). The present study reports the number of cases who underwent a second intervention, nevertheless we have no information as to whether a third or fourth intervention was performed or whether additional surgery was included in the initial protocol or was required for complications related to a previous intervention. A total of 1012 patients underwent a second procedure, representing 36.4% of the total number of patients treated, and being similar to the results of Hosseini *et al*. (33) who reported 41.05% of second interventions. We also found that fistula repair was the most frequently intervention performed in 428 patients (42.29%; 174 females and 254 males) with a median age of 6 years.

The present study included data from 78 surgical missions of Operation Smile including patients from all over Peru. To our knowledge this is the first study to evaluate the clinical and epidemiological profiles of the Peruvian populations attended over 13 years. The collection of data by geographic area and the identification of the most frequent and prevalent patterns of craniofacial anomalies such as cleft lip and palate provides better understanding of the incidence, evolution and magnitude of these conditions and will help to improve the planning of future actions for the care of these patients

## Conclusions

A comprehensive understanding of the target population is crucial to planning preventive and curative strategies in surgical missions. In our study population, cleft lip and palate are more frequent in males compared to females. The most frequent diagnosis was CP, CLP was more frequent on the left side and in males, and the first surgical intervention is aimed at closure of the lip.
